# Manipulation of Dysfunctional Spinal Joints Affects Sensorimotor Integration in the Prefrontal Cortex: A Brain Source Localization Study

**DOI:** 10.1155/2016/3704964

**Published:** 2016-03-07

**Authors:** Dina Lelic, Imran Khan Niazi, Kelly Holt, Mads Jochumsen, Kim Dremstrup, Paul Yielder, Bernadette Murphy, Asbjørn Mohr Drewes, Heidi Haavik

**Affiliations:** ^1^Mech-Sense, Department of Gastroenterology and Hepatology, Aalborg University Hospital, 9000 Aalborg, Denmark; ^2^Centre for Chiropractic Research, New Zealand College of Chiropractic, Auckland 1060, New Zealand; ^3^Centre for Sensory-Motor Interactions (SMI), Department of Health Science and Technology, Aalborg University, 9100 Aalborg, Denmark; ^4^Faculty of Health & Environmental Sciences, Health & Rehabilitation Research Institute, AUT University, Auckland 1010, New Zealand; ^5^Faculty of Health Sciences, University of Ontario Institute of Technology, ON, Canada L1H 7K4

## Abstract

*Objectives*. Studies have shown decreases in N30 somatosensory evoked potential (SEP) peak amplitudes following spinal manipulation (SM) of dysfunctional segments in subclinical pain (SCP) populations. This study sought to verify these findings and to investigate underlying brain sources that may be responsible for such changes.* Methods*. Nineteen SCP volunteers attended two experimental sessions, SM and control in random order. SEPs from 62-channel EEG cap were recorded following median nerve stimulation (1000 stimuli at 2.3 Hz) before and after either intervention. Peak-to-peak amplitude and latency analysis was completed for different SEPs peak. Dipolar models of underlying brain sources were built by using the brain electrical source analysis. Two-way repeated measures ANOVA was used to assessed differences in N30 amplitudes, dipole locations, and dipole strengths.* Results*. SM decreased the N30 amplitude by 16.9 ± 31.3% (*P* = 0.02), while no differences were seen following the control intervention (*P* = 0.4). Brain source modeling revealed a 4-source model but only the prefrontal source showed reduced activity by 20.2 ± 12.2% (*P* = 0.03) following SM.* Conclusion*. A single session of spinal manipulation of dysfunctional segments in subclinical pain patients alters somatosensory processing at the cortical level, particularly within the prefrontal cortex.

## 1. Introduction

Over the past decade, there has been a growing body of evidence to suggest that neural plastic changes occur following chiropractic spinal manipulation [1]. Investigators utilizing techniques such as transcranial magnetic stimulation and somatosensory evoked electroencephalographic (EEG) potentials have suggested that neuroplastic brain changes occur in structures such as the primary sensory cortex, primary motor cortex, prefrontal cortex, basal ganglia, and cerebellum [2–6]. However, the evidence for the involvement of these brain structures is indirect. Although EEG measures neuronal activity directly (with high millisecond time resolution), it has poor spatial resolution, making it difficult to know exactly where in the brain the changes are occurring. Studies with only a few recording EEG electrodes [3, 6] allow investigation of evoked potential amplitudes and latencies and have shown changes in the N30 somatosensory evoked potential (SEP) amplitudes following spinal manipulation, but they do not allow identification of changes in the individual areas of the brain generating the neural activity that underlies the evoked signals.

In recent decades, efforts have been made to improve the spatial resolution of EEG [7]. These methods have successfully been used in a number of SEP studies, generally showing a five-dipole model involving primary and secondary somatosensory cortices, insula, cingulate, and prefrontal cortex [8–10].

With this study, we set out to utilize brain electrical source analysis to explore which brain sources are responsible for changes in N30 amplitude following a single session of spinal manipulation. We hypothesized that a single session of chiropractic spinal manipulation would reduce the N30 amplitude and that this amplitude reduction would be attributed to decreased strength of one or more of the underlying brain sources. Therefore, the aims of this study were to

1. compare amplitudes of N30 potential between baseline and postcontrol intervention (sham chiropractic treatment),
2. compare amplitudes of the N30 potential between baseline and postspinal manipulation session,
3. assess whether there are differences in N30 amplitudes between the control and the spinal manipulation session,
4. assess whether there are differences between the chiropractic and the control sessions in the underlying brain sources.

## 2. Experimental Procedures

### 2.1. Subjects

Nineteen subclinical pain volunteers were included in the study (9 males, 25.6 ± 3.9 years). Subclinical pain (SCP) refers to recurrent spinal ache, pain, or stiffness for which the person has not yet sought treatment [11, 12]. The study was conducted according to the Declaration of Helsinki. The local Ethics Committee (N-20140027) approved the study. The study was conducted in the laboratories at the Department of Gastroenterology and Hepatology, Aalborg University Hospital, Aalborg, Denmark.

### 2.2. SEP Stimulating and Recording Parameters

The EEG signals were recorded with Neuroscan System (version 4.5, El Paso, TX) from 62 scalp electrodes using the extended 10-20 system montage (Quick-Cap International, Neuroscan, El Paso, TX). The subjects were seated comfortably in supine position with eyes open throughout the entire recording. The subjects received electrical stimulations applied to the median nerve at the right wrist to evoke somatosensory potentials. Two trials of 1000 pulses were given in each session: one trial before treatment (control or chiropractic) and one trial after the treatment. The pulses were given at 2.3 Hz stimulation frequency and were of 0.2 ms length. The intensity of the stimulus was modified to be 1 mA above the stimulation intensity that elicited clear twitch of the thumb. The EEG signal was sampled at 10,000 Hz with open online filters.

### 2.3. Experimental Protocol

The subjects were asked to attend three sessions. A screening session for inclusion and exclusion criteria was followed by the two experimental sessions (control and chiropractic) in random order. During the screening session, a chiropractor assessed the subject's spine and history to assure that they fit the criteria for subclinical pain, had no contraindications to receiving spinal manipulation, and displayed the presence of spinal dysfunction. The subjects were excluded from the study if any of the following was true: no evidence of spinal dysfunction was present, they were in current pain, they had sought previous treatment for their spinal issues, or they had contraindications to receiving spinal manipulation.

### 2.4. Interventions

#### 2.4.1. Spinal Manipulation

The entire spine and both sacroiliac joints were assessed for segmental dysfunction (also referred to as vertebral subluxation by many members of the chiropractic profession) and treated where they were deemed necessary by a registered chiropractor with fifteen years of clinical experience. The clinical indicators that were used to assess the function of the spine prior to and after each spinal manipulation intervention included assessing for tenderness to palpation of the relevant joints, manually palpating for restricted intersegmental range of motion, assessing for palpable asymmetric intervertebral muscle tension, and any abnormal or blocked joint play and end-feel of the joints. All of these biomechanical characteristics are used by the chiropractic profession as clinical indicators of spinal dysfunction [13]. All of the spinal manipulations carried out in this study were high-velocity, low-amplitude thrusts to the spine or pelvic joints. This is a standard manipulation technique used by chiropractors and is also referred to as spinal adjustments. The mechanical properties of this type of central nervous system perturbation have been investigated; and although the actual force applied to the subject's spine depends on the therapist, the patient, and the spinal location of the manipulation, the general shape of the force-time history of spinal manipulations is very consistent [14] and the duration of the thrust is always less than 200 milliseconds [15]. The high-velocity type of manipulation was chosen specifically because previous research has shown that reflex electromyographic activation observed after manipulations only occurred after high-velocity, low-amplitude manipulations (as compared with lower-velocity mobilizations) [16]. This manipulation technique has also been previously used in studies that have investigated the neurophysiological effects of spinal manipulation [1].

#### 2.4.2. Control Intervention

As the subjects in this study were naïve to chiropractic care, we provided a sham treatment session. One of the investigators, who was not a chiropractor, therefore simulated a chiropractic treatment session. This included passive and active movements of the subject's head, spine, and body, similar to what was done by the chiropractor who provided the actual chiropractic treatment during the spinal manipulation intervention. Thus, this control intervention involved the subjects being moved into the manipulation setup positions similar to how the chiropractor would normally set up a subject prior to applying the thrust to the spine to achieve the manipulations. The sham treatment provider was particularly careful not to put pressure on any individual spinal segments. Loading a joint, as is done prior to spinal manipulation, has been shown to alter paraspinal proprioceptive firing in anesthetized cats [17] and therefore was carefully avoided by ending the movement prior to end-range-of-motion when passively moving the subjects. No spinal manipulation was performed during any control intervention. This control intervention was intended to act as a sham treatment session as well as to act as a physiological control for possible changes occurring due to the cutaneous, muscular, or vestibular input that would occur with the type of passive and active movements involved in preparing a subject/patient for a manipulation. It also acted as a control for the effects of the stimulation necessary to collect the dependent measures of the study, and for the time required to carry out the manipulation intervention.

### 2.5. Data Analysis

#### 2.5.1. Preprocessing of the Evoked Potentials

The preprocessing of SEP data was done in Neuroscan (version 4.5, El Paso, TX). SEP data were first bandpass-filtered between 1 and 1000 Hz. Then, the raw data were divided into epochs and visually cleaned for artifacts. Baseline and treatment recordings were then compared in order to see whether one recording had more epochs deleted due to artifacts; if this was the case, then the recording that had less epochs deleted was further cleaned by deleting the last few epochs such that the two recordings would have the same number of epochs. This was done to reduce the influence of number of epochs on SEP amplitude. The epochs were then averaged and the noisy channels were interpolated. Finally, data were referenced to the parietal-temporal electrode contralateral to the stimulated arm (TP7) to increase the amplitude of N30 potential at the frontal electrodes.

#### 2.5.2. Amplitude Analysis of N30

Amplitude analysis of the N30 peak was done at the frontal electrode contralateral to the stimulated arm (F3). This electrode was chosen because visual inspection revealed that the N30 peak tended to be the highest at this sight. The amplitude was measured as peak-to-peak from amplitude of the positivity preceding the N30 to the amplitude where N30 was the highest.

#### 2.5.3. Source Localization

Dipolar source modeling was performed in brain electrical source modeling (BESA) (BESA Research 5.3; MEGIS Software GmbH, Gräfelfing, Germany). The potential distributions over the scalp from preset voltage dipoles within the brain were calculated. Then, the agreement between the recorded and calculated field distributions was evaluated. The percentage of data that could not be explained by the model was expressed as residual variance (RV). A spherical three-shell model with an 85 mm radius was used and it was assumed that the brain surface was 70 mm from the centre of the sphere [8]. For both experimental sessions, the model was first created on grand-averages of each baseline recording. Then, these models were applied to their respective posttreatment grand mean files in order to get an idea of whether any changes existed. Then, these models were applied to the individual data. In order to obtain an idea of the number and location of sources, the dipolar models were LORETA-guided. LORETA is a current density method which yields blurred source images. The advantage of LORETA is that no a priori constraints regarding the number and location of sources are required and its accuracy has been proven to be high [18]. Once the dipolar models for all the subjects and sessions were done, the source activation waveforms were exported to MATLAB (version 8.4.0, The Mathworks Inc., Natick, MA, USA) and brain source strengths were computed by means of area under the curve (AUC). The AUC was calculated between 25 and 45 ms after stimulus such that mainly the source strength during the N30 peak was taken into consideration. The model calculated by BESA is a hypothetical one and does not exclude other solutions, but, nevertheless, it can be validated when applicable to individual data and consistent with anatomical and physiological knowledge of identified source areas [19].

#### 2.5.4. Statistics

Descriptive statistics are reported as mean ± SD. To compare data between the control session and chiropractic session, two way repeated measures analysis of variance was used (ANOVA). N30 amplitudes and brain source strengths were compared between the two sessions. If overall significance for any of the ANOVA tests was found, all pairwise multiple comparison procedures (Holm-Sidak method) were done in order to see which variables were significantly different. The software package SigmaStat version 3.0 (SPSS Inc., Chicago, IL, USA) was used for statistical analysis.

## 3. Results

SEPs were successfully recorded in all 19 volunteers. The stimulation intensity used during session one was 8.7 ± 3.7 mA and during session two it was 7.9 ± 3.1 mA. There were no significant differences between the two sessions (*P* = 0.5).

Upon questioning, after both sessions were conducted, and despite the subjects being totally naïve to chiropractic, the majority of the subjects guessed correctly in which session they received the actual spinal manipulations and which was the sham. When asked why they were sure which session was real, the majority noted they could feel that the chiropractic session actually changed the way their body felt and functioned.

### 3.1. N30 Amplitude Results

**Figure 1:**
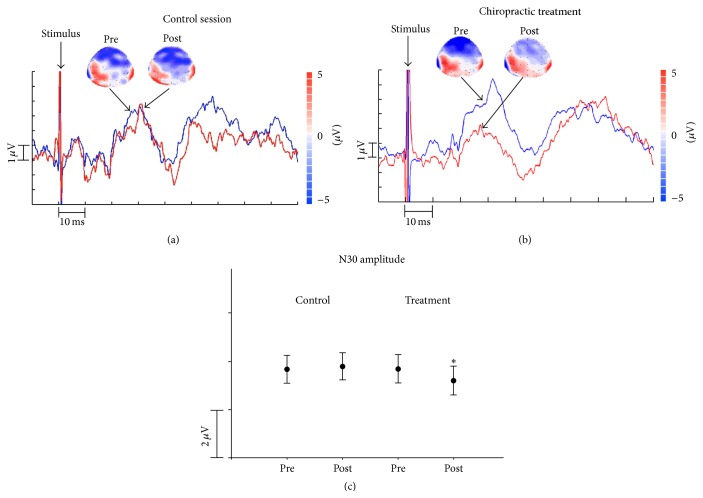
(a) Waveforms and topographies of baseline against the recording after control treatment. Blue waveform is baseline. Topographies are N30 topographies. This is a plot of one representative subject. (b) Waveforms and topographies of baseline against the recording after chiropractic treatment. Blue waveform is baseline. Topographies are N30 topographies. This is a plot of one representative subject. (c) Error bars for N30 amplitude. *∗* represents significant difference.

There was a significant postintervention difference between the two groups. Post hoc analysis revealed that the N30 amplitude was reduced in the spinal manipulation group following the treatment (*P* = 0.02), while it remained stable in the control group (*P* = 0.4). Please see Figure 1.

### 3.2. Brain Source Localization

**Figure 2:**
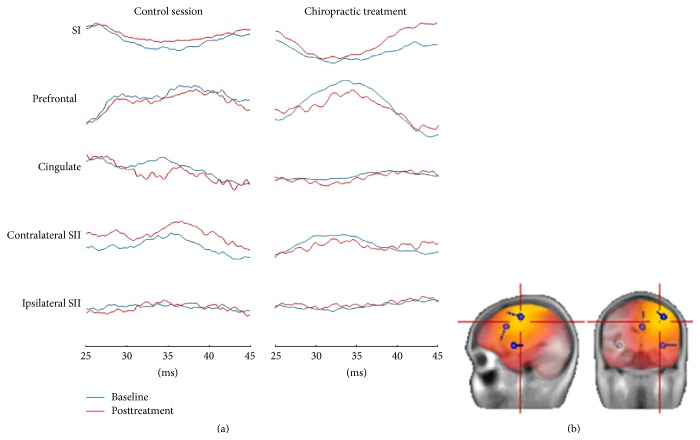
Source localization and activity. (b) are the dominant sources and (a) are their waveforms of activity. The plot is of one representative subject.

One of the subjects had poor signal-to-noise ratio and the peaks could not be identified when looking at all the electrodes simultaneously; this subject was excluded from source localization analysis. For the remaining subjects, the time interval between 20 and 60 ms with respect to stimulation was chosen for brain source analysis. The LORETA solution revealed four distinct solutions during this time interval: contralateral primary somatosensory cortex (SI), prefrontal cortex, cingulate, and bilateral secondary somatosensory cortex (SII). Therefore, we assumed a 4-source solution in these brain areas. We placed the first dipole in contralateral SI, a second dipole in contralateral prefrontal cortex, third dipole in cingulate cortex, and fourth dipole in contralateral SII, and fifth dipole had a symmetry constraint to the contralateral SII dipole based on the symmetry assumption of the two hemispheres [20]. Once all the dipoles were fixed in their position, the orientations were allowed to move freely and the solution shown in Figure 2 was obtained. This model was then applied to all the individual data and the final solution shown in Table 1 was obtained. It can be seen that the prefrontal cortex tended to have the highest strength during this time interval. The RV values were below 10% for all the subjects/sessions.

**Figure 3:**
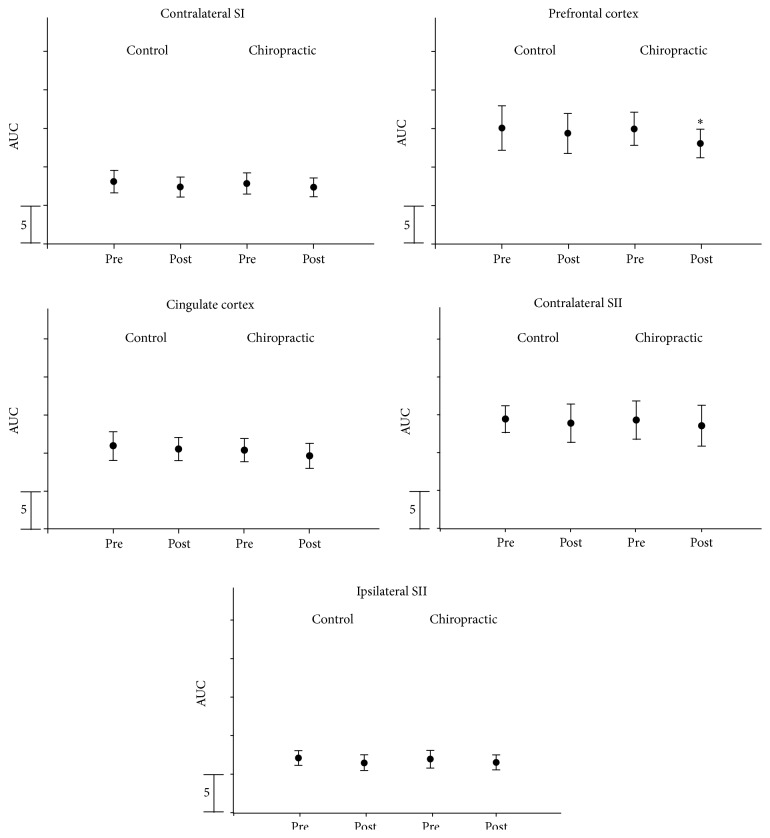
AUC error bars for each of the sources. *∗* represents significant difference.

Source strength analysis revealed that chiropractic treatment reduced the strength of the prefrontal source (*P* = 0.03), while all the other strengths remained stable (*P* > 0.2). Please see Figure 3.

## 4. Discussion

This study resulted in two major findings. Firstly, this study reproduced previous findings of SEPs studies that have shown that adjusting dysfunctional spinal segments alters early sensorimotor integration (SMI) of input from the upper limb (as evidenced with a decrease in N30 SEP complex amplitudes) [3, 6, 21]. The second major finding of this study was that we were able to show, using dipole source localization, that this change in SMI that occurs after spinal manipulation predominantly happens in the prefrontal cortex.

### 4.1. The N30 Peak

The N30 SEP peak has been shown to have multiple neural generators including primary sensory cortex, basal ganglia, thalamus, premotor areas, and primary motor cortex [22–28]. The frontal N30 peak is therefore thought to reflect early SMI [29, 30].

The N30 component is the most vulnerable SEP component to the gating effect that occurs during voluntary muscle contraction [31] and is known to occur even when subjects only mentally imagine moving muscles [31, 32]. Despite early theories to the contrary [26, 27, 33], the supplementary motor area (SMA) has been ruled out as a source for the N30 in studies with intracortical electrodes stereotactically implanted in the frontal lobe of epileptic patients, as they demonstrated that no early SEP was generated in pre-SMA or SMA-proper in the first 50 ms after stimulation [34, 35].

Research has shown that the frontal N30 component has independent cortical generators with a separate thalamocortical input [25, 36]. Intracortical human recordings have also shown afferent information following median nerve stimulation project directly to the primary motor cortex [36], as has been documented previously in primates [37, 38]. The frontal N30 component is therefore subject to more complex inputs than those flowing on from the parietal N20 generator (i.e., S1) alone.

The primary motor cortex is one of the known sources of the N30 [28]. Waberski et al. [28] applied dipole source localization and current density reconstruction within individual realistically shaped head models and demonstrated that the source of the N30 peak resided within the precentral motor cortex (area 4). More recently, Cebolla et al. [30] have used swLORETA (standardized weighted Low Resolution Brain Electromagnetic Tomography) taking into account both phasic and oscillatory generators to determine the neural generators of the N30. They demonstrated that the N30 is generated by network activity in the motor, premotor, and prefrontal cortex, adding further weight to its role as a marker of neural processing relevant to SMI.

Several studies have linked the basal ganglia with the N30 SEP component [39–41] and consider it to reflect activity in basal ganglionic thalamocortical loops linking primary motor cortex, premotor cortex, and prefrontal cortex [41]. There is a number of clinical research studies to support this view since the N30 peak amplitude is decreased in Parkinson's disease patients [39, 40, 42], and deep brain stimulation of basal ganglia nuclei such as the subthalamic nucleus can produce a selective increase of the N30 amplitude, thought to be due to improved SMA functional activity [39]. Furthermore, blocking the neuromuscular junction in Parkinson's disease patients also increases the N30 amplitude as well as reducing the rigidity of their muscles [40].

The current study adds to previous work by not only confirming that spinal manipulation of dysfunctional joints decreases the N30 SEP peak amplitude but also demonstrating that this decrease occurs predominantly in one of the known neural generators of N30, that is, the prefrontal cortex.

### 4.2. The Prefrontal Cortex and Executive Function

Our current study findings confirmed that spinal manipulation of dysfunctional spinal segments reduces the N30 SEP peak amplitude and using dipole source localization demonstrated that this change is taking place in the prefrontal cortex. This suggests that, at least in part, the mechanisms by which spinal manipulation improves performance are due to a change in function at the prefrontal cortex. The prefrontal cortex is known to play a vital role in SMI and is also responsible for a number of other functions. The prefrontal cortex is known to be a key structure responsible for the performance of what is known as “executive functions” [43, 44]. Executive function is the mechanism by which the brain integrates and coordinates the operations of multiple neural systems to solve problems and achieve goals based on the ever-changing environment around us [44, 45]. Executive function is considered to be a product of the coordinated operation of various neural systems and is essential for achieving any particular goal. The prefrontal cortex is believed to be the main brain structure responsible for enabling this coordination and control. It requires planning a sequence of subtasks to accomplish a goal, focusing attention on relevant information as well as inhibiting irrelevant distractors, being able to switch attention between tasks, monitoring memory, initiation of activity, and responding to stimuli [44–46]. A change in prefrontal activity following chiropractic care may therefore explain and/or link some of the varied improvements in neural function previously observed in the literature, such as improved joint position sense error [47], reaction time [48], cortical processing [3, 48], cortical sensorimotor integration [3, 5, 6], reflex excitability [49–52], motor control [5, 53], and lower limb muscle strength [54].

To accomplish the coordinated operations of multiple neural systems and structures, the prefrontal cortex must monitor the activities in other cortical and subcortical structures and control and integrate their operations by sending command signals in a so-called “top-down” manner. This is a complex operation, and the importance of this monitoring, integration, and coordination is highlighted in studies where damage to the prefrontal cortex has been shown to impair the ability to create new and adaptive action programs or choose the best among several equally probable alternatives, despite such individuals displaying normal IQs in most psychological tests, having normal long-term memory functions, and exhibiting normal perceptual, motor, and language skills [43]. The change in prefrontal cortex as seen in this study therefore suggests that the altered input from dysfunctional joints that leads to altered processing of somatosensory inputs can influence processing of somatosensory information by the prefrontal cortex. Chiropractic care, by treating the joint dysfunction, appears to change processing by the prefrontal cortex. This suggests that chiropractic care may as well have benefits that exceed simply reducing pain or improving muscle function and may explain some claims regarding this made by chiropractors [55, 56].

### 4.3. Study Considerations

Although the change in N30 due to chiropractic treatment is an important finding, it is not clear how long this finding lasts. To date, some of the authors of this study have shown that the N30 changes on average are present for at least 20–30 minutes after spinal manipulation [3]. For some subjects, the changes were still evident at 30 minutes after spinal manipulation and we have not yet followed up for longer than 30 minutes, due to the length of the study as is.

The authors of this study assume that since spinal manipulation is known to reduce pain and improve function in clinical trials [57–59], the observed reduction of the N30 amplitudes reflects a beneficial change. However, it should be noted that reduced N30 SEP peak amplitudes have been found in the literature in pathological condition such as Parkinson's disease [42]. This should therefore be followed up in future studies.

The calculated dipolar sources from the surface EPs should not be seen as precise indicators of where brain activity is, but more as an estimate of where dominant activity is occurring (the so-called “center of gravity”). Brain source localization, therefore, allows us to estimate where the dominant brain activity is occurring due to sensory stimulation and how this activity is modified following chiropractic treatment.

## 5. Conclusion

This study has reproduced the findings of previous SEP studies that have shown that adjusting dysfunctional spinal segments alters early SMI of input from the upper limb (as evidenced with a decrease in N30 SEP complex amplitudes). It also expands on this finding by using dipole source localization to show that this change in SMI that occurs after spinal manipulation predominantly happens in the prefrontal cortex. Hence, the mechanisms behind pain relief following spinal manipulation in low level pain patients are likely due to improved SMI and appropriate motor control, as this is the key function of the prefrontal cortex.

## Figures and Tables

**Table 1:** Brain source coordinates and their areas under curve.

	Control					Chiropractic				
	*X*	*Y*	*Z*	AUC Pre	AUC Post	*X*	*Y*	*Z*	AUC Pre	AUC Post
SI	−44 ± 3	−15 ± 3	34 ± 5	8 ± 6	7 ± 5	−43 ± 3	−16 ± 11	39 ± 6	8 ± 6	7 ± 5
Prefrontal	−34 ± 5	3 ± 8	32 ± 8	15 ± 12	14 ± 11	−33 ± 5	7 ± 11	28 ± 7	15 ± 9	13 ± 8^*∗*^
Cingulate	4 ± 9	0 ± 3	15 ± 4	11 ± 8	11 ± 6	4 ± 8	−2 ± 3	22 ± 8	10 ± 7	10 ± 7
Cont. SII	−37 ± 3	1 ± 2	−4 ± 4	14 ± 7	14 ± 11	−38 ± 5	1 ± 2	−2 ± 6	14 ± 10	13 ± 12
Ips. SII	37 ± 3	1 ± 2	−4 ± 4	7 ± 4	7 ± 4	38 ± 5	1 ± 2	−2 ± 6	7 ± 5	7 ± 4

Significant differences are shown by *∗*. AUC: area under curve; SI: primary somatosensory cortex; Cont. SII: contralateral secondary somatosensory cortex; Ips. SII: ipsilateral somatosensory cortex.
